# 

**Published:** 1887-07-16

**Authors:** 


### CONTENTS.

**Philanthropy as a Career** - - - - - 257
**Words of Consolation.**—XLI. The Work of the Holy Ghost - - - - - - - - 258
**Flower Services and Hospitals** - - - - 258
**The Story of the Hospitals.**—Guy's Hospital - 259
**The Doctor's Pulpit.**—III. Nerves: their Uses and Troubles - - - - - - - - 261
**Doctors in Fiction** - - - - - - 262
**Chats about New Books** - - - - - 263
**Notes and News.**—Miscellaneous Hospital Items.—Opening of the National Hospital, Bloomsbury, by the Duchess of Albany.—Bazaars, Festivals, etc., in Aid of the Hospitals.—Vacancies.—Medical Relief and the Charity Organisation Society.—The Miners' Hospital.—Convalescent Home or Hospital.—The Mary Wardell Convalescent Home.—Prize Day at St. Thomas's Medical School.—The Nottingham General Hospital.—New Hospital at Bournemouth.—The Bath Mineral Water Hospital.—Hospital Saturday Fund Returns.—The Rugby Provident Dispensary.—Artificial Respiration for the Drowned.—The Worcester Infirmary and the Monthly Visitors.—Annual Meetings and Reports, etc. - 264
**Annotations.**—The Hospitals Association and its Future Work.—Cook your Milk.—Who Nurses the Sick Nurse?—Bald Heads - - - - - - - 267
**The Medical Treatment of Women in India.** By a Supporter of the Countess of Dufferin's Fund - - - - - - - - 268
**A Friendly Alias.**—Chapters I and II - - - - 269
**In- and Out-Patients' Hospital Diary** - 272
**The Children's Corner.**—Puzzles, Answers, etc. - vi
**Amusements with Profit** - - - - - vi

